# HIV-1 Transmission within Marriage in Rural Uganda: A Longitudinal Study

**DOI:** 10.1371/journal.pone.0055060

**Published:** 2013-02-04

**Authors:** Samuel Biraro, Eugene Ruzagira, Anatoli Kamali, James Whitworth, Heiner Grosskurth, Helen A. Weiss

**Affiliations:** 1 Medical Research Council (MRC)/Uganda Virus Research Institute (UVRI) Uganda Research Unit on AIDS, Entebbe, Uganda; 2 MRC Tropical Epidemiology Group, Department of Infectious Disease Epidemiology, London School of Hygiene and Tropical Medicine, London, United Kingdom; 3 Wellcome Trust, London, United Kingdom; University of Ottawa, Canada

## Abstract

**Background:**

Early initiation of antiretroviral therapy reduces risk of transmission to the uninfected partner in HIV discordant couples, but there are relatively little observational data on HIV transmission within couples from non-trial settings. The aims of this paper are to estimate HIV incidence among HIV discordant couples using longstanding observational data from a rural Ugandan population and to identify factors associated with HIV transmission within couples, including the role of HSV-2 infection.

**Methods:**

Using existing data collected at population-wide annual serological and behavioural surveys in a rural district in southwest Uganda between 1989 and 2007, HIV discordant partners were identified. Stored serum samples were tested for HSV-2 serostatus using the Kalon ELISA test. HIV seroconversion rates and factors association with HIV seroconversion were analysed using Poisson regression.

**Results:**

HIV status of both partners was known in 2465 couples and of these 259 (10.5%) were HIV serodiscordant. At enrolment, HSV-2 prevalence was 87.3% in HIV positive partners and 71.5% in HIV negative partners. Of the 259 discordant couples, 62 converted to HIV (seroconversion rate 7.11/100 PYAR, 95%CI; 5.54, 9.11) with the rate decreasing from 10.89 in 1990–1994 to 4.32 in 2005–2007. Factors independently associated with HIV seroconversion were female sex, non-Muslim religion, greater age difference (man older than woman by more than 15 years), higher viral load in the positive partner and earlier calendar period. HSV-2 was not independently associated with HIV acquisition (HR 1.62, 95%CI; 0.57, 4.55) or transmission (HR 0.61, 95%CI; 0.24, 1.57). No transmissions occurred in the 29 couples where the index partner was on ART during follow up (872 person-years on ART).

**Discussion:**

HIV negative partners in serodiscordant couples have a high incidence of HIV if the index partner is not on antiretroviral therapy and should be provided with interventions such as couple counselling, condoms and antiretroviral treatment.

## Background

In generalised HIV epidemics in sub-Saharan Africa, a substantial proportion of new HIV infections occur in cohabiting couples [1]. For example, studies from Rwanda and Zambia estimate that 55–93% of new heterosexually acquired HIV infections occurred within stable partnerships [2], although lower estimates (10–52%) have been reported [3]. In Uganda, a modes of transmission study estimated that among adults aged 15 to 49 years, 43% of new HIV infections in 2008 occurred in monogamous relationships [4].

About 50% of married or cohabiting HIV positive individuals in stable partnerships in East and Southern Africa are in an HIV-serodiscordant relationship [5]. Among married or cohabiting couples in the general population in rural Uganda, 5–7% were estimated to be HIV serodiscordant [6], [7]. HIV transmission rates in serodiscordant partnerships are high, ranging from 3.7 to 19.0 per 100 person years at risk (PYAR) [1], [6], [8]–[12].

HIV serodiscordant couples are suitable for studying potential interventions for HIV prevention and have been studied in clinical trials evaluating HSV-2 suppressive treatment [13], vaginal microbicides [14] and prophylaxis with HAART [15]. HAART has been shown to reduce risk of transmission to the uninfected partner by 96% [15], however it is unlikely to become quickly available to populations in need. Studies among HIV serodiscordant couples can provide insights into the dynamics of HIV transmission that may assist with future interventions [16]. However there are few studies of HIV serodiscordant couples with longstanding, observational data. In this paper, we analyse observational data on HIV and HSV-2 status of partners in a rural Ugandan community collected from 1989 to 2007, to study the association of HSV-2 infection with HIV transmission in serodiscordant couples. The aims of this paper are i) to estimate HIV incidence among HIV discordant couples in this rural Ugandan population from 1989 to 2007; ii) to assess the role of HSV-2 infection on HIV transmission within couples and iii) to identify factors associated with HIV transmission within couples.

## Methods

### Setting

The study population comprises approximately 20,000 residents of 25 neighbouring villages in southwestern Uganda. The community is stable and homogeneous, with most people from the Baganda tribe (73%), and 15% of Rwandese origin. Religious affiliation is mostly Christian, with a significant Muslim minority (28%). HIV prevalence is high (7.7% in 2005) [17].

Details of the cohort and annual HIV serosurvey have been published previously [18]–[20]. In brief, household surveys of socio-demographic and behavioural characteristics and HIV serostatus of consenting participants aged 13 years and above have been conducted annually since 1989, with all residents eligible for inclusion. The average annual participation rate is 60%–65%. The survey consists of an annual door-to-door census, followed by a sero-behavioral survey in which consenting residents are interviewed at home and asked to provide a blood sample for HIV testing.

### Participants

The present analyses use data collected at annual surveys between 1989 and 2007. At each survey, household members were assigned a code indicating their relationship to others in the household. In addition, each married participant was asked to name their spouse. These data allowed for retrospective linkage of partners who participated in the surveys as individuals. The study included couples where both spouses were aged 18 to 59 years.

### Laboratory Methods

HSV-2 status was determined using the Kalon ELISA assay [21]. HIV was determined using two ELISA tests confirmed with Western Blot in case of first time positives or discordant ELISA [22].

### Statistical Methods

Statistical analyses were performed using Stata version 11.0 (StataCorp, Texas USA). Couples identified in the database were defined as concordant negative if both spouses were HIV negative, discordant if one spouse was HIV positive and the other was HIV negative, concordant positive if both were HIV positive, incomplete/unknown if the HIV status of one or both was not known. When an individual did not have an HIV test result, the HIV status was imputed as follows: a participant testing positive at one survey but missing a result on a subsequent survey at which they were present was imputed as positive, while one testing negative at a survey but missing a result on an earlier survey at which they were present was imputed as negative.

Missing HSV-2 data were imputed using the same method. In addition, to increase precision of the estimated HSV-2 prevalence and to reduce bias in analyses for the effect of HSV-2 on HIV seroconversion in discordant couples, missing HSV-2 status was further imputed using multiple imputation [23], which assumes that data are missing at random. Variables included in the imputation model were gender, age, HIV status, and HSV-2 status of either partner.

HIV incidence rates and 95% confidence intervals (CI) were estimated using Poisson regression. Follow-up time started at the visit when couples were first seen as serodiscordant and ended either at estimated seroconversion date, or the date last seen (for couples who did not seroconvert). If the couple was not seen on the same date, the date for HIV test results from the HIV negative partner was used. The date of HIV seroconversion was estimated as the midpoint of the last negative and first positive test.

Factors associated with HIV incidence were estimated using Poisson regression with random-effects to allow for within-person clustering of men included with more than one partner. There was a-priori interest in potential interactions between age and sex of the susceptible partner and of the index partner, and these were examined using the Wald test. However, as there was no evidence of interaction, primary analyses were not stratified by sex or age. Separate models were used to analyse characteristics for the negative partner and the positive partner. A hierarchical conceptual framework approach was used [24]. Socio-demographic and behavioural factors (age, sex, religion, level of education, tribe, extramarital partners in the previous year, age difference between spouses, polygamy, calendar period) that were significant at p≤0.2 on univariable analyses were entered in a multivariable model and retained if they remained significant at p≤0.2. In this community where circumcision is not culturally practised, Muslim religion is a proxy for male circumcision. Biological factors (HSV-2 status, CD4 count, plasma viral load, use of highly active antiretroviral therapy (HAART) in the HIV positive partner) were then added to this core socio-demographic model one at a time, and those with p≤0.2 were retained. These clinical data were only available for a sub-group of the study participants who were participating in clinical studies [25]. As HSV-2 was the primary factor of interest, it was retained in the model irrespective of significance level. Finally, factors which remained significant at p≤0.05 after adjustment for all others were considered independently associated with seroconversion. To better understand the factors associated with HIV seroconversion, additional analyses were stratified by sex of the positive partner. For ordered categorical variables such as age group, trend was determined and p-value for trend reported if significant.

### Ethics Statement

The study obtained ethics approval from the scientific and ethics review boards from the London School of Hygiene and Tropical Medicine, Uganda Virus Research Institute and the Uganda National Council for Science and Technology. The survey population was actively encouraged to test for HIV infection, using the freely available testing and counselling services, including couple counselling, and condom provision. The present study used existing data and therefore did not offer couple counselling. Beginning with 2004 when HAART became available in this population, eligible study participants were started on treatment according to the Uganda national guidelines.

## Results

### Study Population

**Figure 1:**
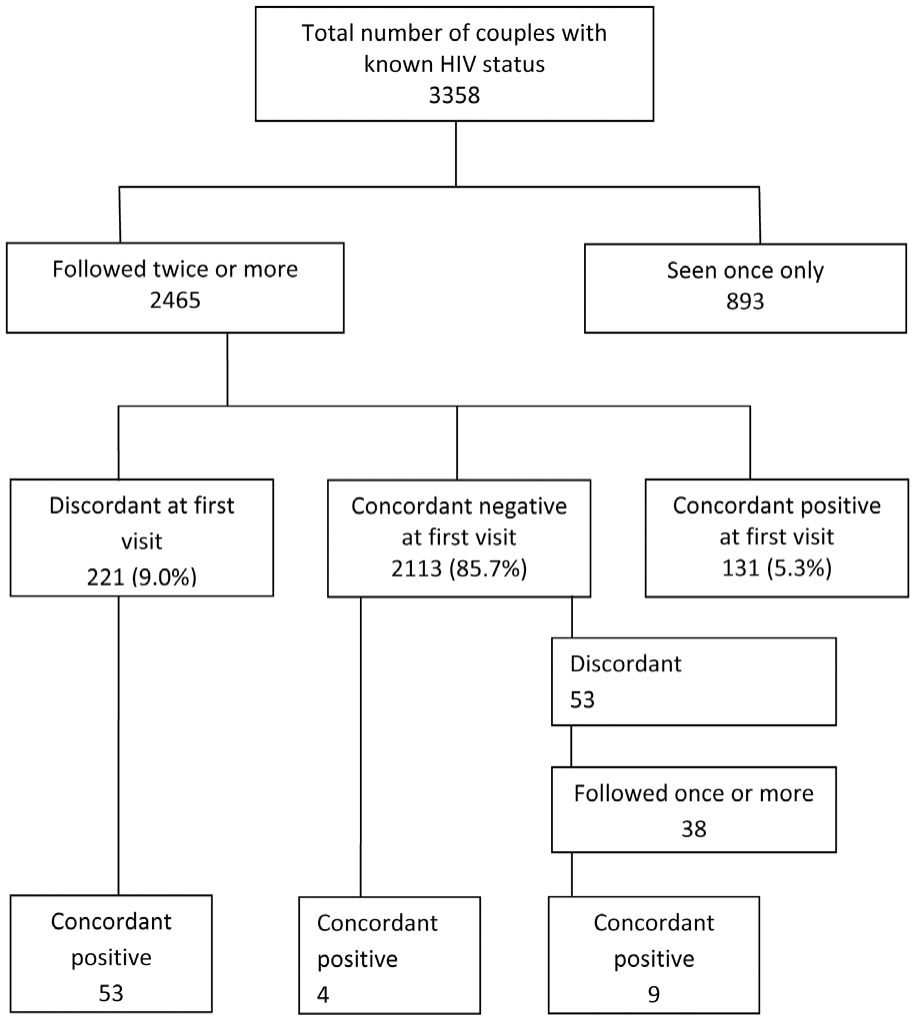
Identification of serodiscordant couples in the General Population Cohort.

From 1989 to 2007, a total of 4,480 couples were seen at least once, with a total of 22,782 visits at which both partners were seen. Of these 3,358 couples (74.9%) had known HIV status at one or more visits and 2,465 of these (73.5%) were seen twice or more (Figure 1). Of these 2,465 couples, 2,113 (85.7%) were concordant negative at first visit with known HIV status, 221 (9.0%) were discordant and 131 (5.3%) were concordant positive (Figure 1). Of the 893 couples seen only once, 158 (17.7%) had been seen for the first time during 2007– the last year of inclusion into this analysis, 19 (2.1%) became ineligible for this study because of ageing whilst data were not available for the remainder (63.2%). The distribution of HIV status among those seen only once differed from those followed more than once, with more discordant (15.1%) and concordant positive (9.2%) couples (p<0.001). Participants seen only once were also more likely to be female and in-migrants, but there were no differences by age, religion, tribe or HSV-2 status (data not shown).

Subsequent analyses focus on the 259 HIV serodiscordant couples (221 couples who were serodiscordant at their first visit and 38 couples who were initially concordant negative but became serodiscordant during follow up; Figure 1). Median follow-up time overall was 2.83 years (interquartile range (IQR): 1.07, 4.41), and 62 couples (23.9%) seroconverted to concordant positive (53 couples who were serodiscordant at the first visit, and 9 couples who became serodiscordant during follow-up). In addition 4 seroconversions occurred among couples who were initially concordant negative and both partners seroconverted between the same two visits (Figure 1).

### Baseline Characteristics of HIV Serodiscordant Couples followed for Seroconversion

The male was the HIV positive partner in 140 (54.1%) of couples. The median age was 33 years (IQR: 27, 39) for males and 26 years (IQR: 21, 32) for females, and was similar by HIV status (p = 0.70). Approximately half (49%) of female HIV positive participants were in-migrants, compared with just 19% of male HIV positive participants (p<0.001). Male HIV negative participants were most likely to be Muslim (33%), compared with 15% of male HIV positive participants. Female HIV positive participants had the lowest levels of education (18% with no education), and male HIV positive participants had the highest levels (6% with no education).

Of the 259 HIV positive partners, 189 (73.0%) had known HSV-2 status at baseline. Of these, 156 were HSV-2 positive (HSV-2 prevalence = 83.7%; 95%CI: 81.7–85.7%). Among the 259 HIV negative partners, 209 (80.7%) had known HSV-2 status, and 148 were HSV-2 positive (HSV-2 prevalence = 71.5%; 95%CI: 69.0–73.9%). Among the 162 couples where both partners had known HSV-2 status at baseline, 13 (8.0%) were HSV-2 concordant negative, 97 (59.9%) were HSV-2 concordant positive, and 52 (32.1%) were HSV-2 discordant at baseline. Among the 52 who were HSV-2 discordant, the HIV index partner was HSV-2 positive in 65.4%. HSV-2 prevalence was higher in females than males among HIV negative (OR = 2.15, 95% CI: 1.52–3.04) and HIV positive participants (OR = 1.32, 95% CI: 0.82, 2.12).

### HIV Seroconversion Rates by Age and Sex

HIV seroconversion occurred in 62/259 (23.9%) partners (incidence rate = 7.11/100 PYAR; 95%CI: 5.54–9.11). HIV incidence was two times higher in females than in males (HR = 2.02, 95% CI: 1.06–3.83; Table 1). The median age at seroconversion was higher in males (40 years, IQR: 30–46 years) than in females (28 years, IQR: 23–33 years; p<0.001). Among females, HIV incidence decreased with age, from 13.03/100 PYAR amongst those aged 18–24 years to 5.23/100 PYAR amongst those aged 40–59 years although it was based on small numbers (p-value = 0.34). There was no evidence of difference in HIV incidence with age among men (p-value = 0.62).

**Table 1:** Rates of HIV seroconversion and transmission among HIV serodiscordant couples and crude hazard ratios for effect of partners’ characteristics.

	HIV negative partner				HIV positive partner			
	Cases/PYAR	‡Rate/1000 PYAR [95% CI]	HR††† [95% CI]	p-value	Cases/PYAR	‡Rate/1000 PYAR [95% CI]	HR††† [95% CI]	p-value
*Partner characteristics*								
***Sex***				p = 0.03				p = 0.03
**Male**	19/382	4.97 [4.14, 5.98]	1		43/490	8.77 [7.76, 9.90]	1	
**Female**	43/490	8.77 [7.76, 9.90]	2.02 [1.06, 3.83]		19/382	4.97 [4.14, 5.98]	0.49 [0.26, 0.94]	
***Age***				p = 0.5				p = 0.6
**18–24**	16/145	11.06 [9.06, 13.51]	1		5/109	4.60 [3.22, 6.58]	1	
**25–29**	13/168	7.74 [6.19, 9.66]	0.79 [0.35, 1.76]		11/154	7.15 [5.62, 9.09]	1.89 [0.60, 5.99 ]	
**30–39**	20/356	5.61 [4.69, 6.71]	0.57 [0.27, 1.22]		31/389	7.97 [6.91, 9.21]	2.05 [0.73, 5.81]	
**40–59**	13/204	6.38 [5.11, 7.97]	0.59 [0.26, 1.38]		15/221	6.78 [5.51, 8.34]	1.83 [0.59, 5.76]	
***Residence***				p = 0.19				p = 0.4
**Resident**	57/757	7.53 [6.77, 8.37]	1		56/765	7.32 [6.57, 8.14]	1	
**In-migrant**	5/115	4.33 [3.03, 6.21]	0.52 [0.20, 1.37]		6/107	5.60 [4.04, 7.77]	0.69 [0.28, 1.68]	
***Religion***				p = 0.02				p = 0.08
**Christian**	56/679	8.25 [7.41, 9.18]	1		54/679	7.95 [7.13, 8.87]	1	
**Muslim**	6/193	3.10 [2.24, 4.30]	0.33 [0.13, 0.84]		8/194	4.13 [3.11, 5.49]	0.46 [0.19, 1.18]	
***Education***				p = 0.6				p = 0.9
**None**	8/80	9.94 [7.49, 13.19]	1		6/77	7.75 [5.59, 10.75]	1	
**Some primary**	44/652	6.75 [5.98, 7.61]	0.69 [0.28, 1.71]		43/585	7.35 [6.51, 8.31]	1.07 [0.40, 2.83]	
**Post-primary**	10/140	7.15 [5.55, 9.20]	0.59 [0.19, 1.83]		13/210	6.18 [4.95, 7.72]	0.89 [0.29, 2.75]	
***Tribe***				p = 0.3				p = 0.14
**Muganda**	45/695	6.47 [5.75, 7.29]	1		44/698	6.31 [5.59, 7.12]	1	
**Other**	17/177	9.58 [7.89, 11.64]	1.38 [0.70, 2.70]		18/175	10.29 [8.52, 12.42]	1.64 [0.84, 3.18]	
***Extramarital partners***				p = 0.6				p = 0.3
**Yes**	58/794	7.30 [6.57, 8.11]	1		56/735	7.62 [6.85, 8.48]	1	
**No**	4/78	5.12 [3.43, 7.64]	0.73 [0.24, 2.19]		6/137	4.37 [3.15, 6.05]	0.55 [0.21, 1.44]	
								
***HAART in index partner***								p<0.001
**No**					62/843	7.35 [6.64, 8.14]	1	
**Yes**					0/29	0	2.3e-13 [0,.]	
***CD4 count*** †								p = 0.9
**0 to 200**					1/22	4.50 [2.022, 10.02]	1	
**201 to 349**					3/41	7.36 [4.64, 11.69]	1.78 [0.16, 19.75]	
**350 to 499**					2/37	5.41 [3.08, 9.53]	1.49 [0.11, 19.87]	
**500+**					5/72	6.96 [4.87, 9.96]	1.56 [0.15, 15.73]	
***Viral Load*** ††								p = 0.11
**<10,000**					1/59	1.71 [0.77, 3.80]	1	
**10,000 to 49,999**					2/49	4.12 [2.34, 7.26]	2.99 [0.23, 39.21]	
**50,000+**					5/39	12.74 [6.81, 18.22]	9.79 [0.97, 98.34]	
***HSV-2 status***				p = 0.13				p = 0.6
**Negative**	4/107	4.08 [2.88, 5.76]	1		5/43	9.55 [6.75, 13.51]	1	
**Positive**	42/612	7.53 [6.76, 8.40]	2.25 [0.78, 6.44]		41/668	6.84 [6.13, 7.62]	0.74 [0.23, 2.39]	
*Couple characteristics*								
***Polygamy***				p = 0.6				
**No**	52/709	7.43 [6.65, 8.30]	1					
**Yes**	10/173	5.79 [4.49, 7.46]	0.82 [0.37, 1.82]					
***Man older by***				p = 0.009				
**< = 15**	50/780	6.41 [5.72, 7.17]	1					
**16+**	12/92	13.00[10.32,16.39]	3.32 [1.36, 8.15]					
***Period in time***				p = 0.15				
**1990–1994**	25/229	10.89 [9.28, 12.78]	1					
**1995–1999**	9/146	6.18 [4.73, 8.07]	0.57 [0.26, 1.29]					
**2000–2004**	19/289	6.57 [5.47, 7.89]	0.66 [0.34, 1.29]					
**2005–2007**	9/208	4.32 [3.31, 5.64]	0.40 [0.18, 0.92]					

† Based on 57 couples where positive partner had at least one CD4 count result.

†† Based on 52 couples where positive partner had at least one Viral load result.

††† Account for clustering for polygamy.

‡ Seroconversion rates calculated from imputed data, but actual number of cases and PYAR presented.

### Factors Associated with HIV Seroconversion

HIV incidence was lowest when the HIV negative partner was Muslim rather than Christian (RR = 0.33, 95%CI 0.13–0.84), and was higher in couples where the man was more than 15 years older than the woman (RR = 3.32, 95%CI 1.36–8.15; Table 1). There was little association of HIV incidence with other socio-demographic characteristics (Table 1), although weak evidence of a decrease in incidence with calendar time, from 10.89/100 PYAR in 1990–1994 to 4.32/100 PYAR in 2005–2007 (p-trend = 0.74).

HIV viral load was associated with increased risk of transmission (RR = 9.79, 95%CI 0.97–98.34 for those with viral load >50,000 copies/mL versus <10,000 copies/mL; Table 1). There were no seroconversions among the 29 couples in which the index partner was on HAART. CD4 data were available for very few participants and for few visits.

HSV-2 infection status of the negative partner increased the risk of HIV seroconversion two-fold, although this was not statistically significant (RR = 2.25, 95%CI: 0.78–6.44; Table 1). Similar results were seen using the observed (non-imputed) data for HSV-2 (RR = 1.84, 95%CI: 0.66–5.13). Among women, there was no evidence of an association between HSV-2 infection and HIV incidence (RR = 1.03, 95%CI 0.29–3.64). Among men, the HIV incidence was 5.78/100 PYR in HSV-2 positive men, and there were no seroconversions in the 34 HSV-2 negative men.

In multivariable analyses of risk factors for HIV acquisition in the HIV negative partner, HIV incidence was independently associated with female sex and non-Muslim religion (Table 2). Similarly, in the model for HIV positive partners, transmission rates were independently associated with male sex and non-Muslim religion. In both models HIV seroconversion increased when the man was older by >15 years and decreased with period in time (Table 2). The association with increasing viral load persisted, but was not statistically significant. There was little evidence of increased risk of HIV seroconversion with HSV-2 seropositivity, either among HIV negative partners (adjusted RR = 1.62, 95%CI 0.57–4.55) or among HIV positive partners (adjusted HR = 0.61, 95%CI 0.24–1.57) (Table 2).

**Table 2:** Factors associated with HIV seroconversion and transmission in serodiscordant couples: multivariable analyses.

	HIV negative partner†Adjusted HR [95% CI]		HIV positive partner†Adjusted HR [95% CI]	
***Sex***		p = 0.03		p = 0.03
**Male**	1		1	
**Female**	1.83 [1.06, 3.18]		0.55 [0.32, 0.95]	
***Religion***		p = 0.001		p = 0.006
**Christian**	1		1	
**Muslim**	0.27 [0.11, 0.68]		0.36 [0.16, 0.80]	
***Viral Load***				p = 0.25
**<10,000**			1	
**10,000 to 49,999**			2.18 [0.19, 24.55]	
**50,000+**			5.85 [0.68, 50.60]	
***HSV-2 status***		p = 0.3		p = 0.4
**Negative**	1		1	
**Positive**	1.62 [0.57, 4.55]		0.61 [0.24, 1.57]	
***Man older by***		p<0.001		p = 0.04
**< = 15**	1		1	
**16+**	3.68 [1.83, 7.4]		3.13 [1.55, 6.31]	
***Period in time***		p = 0.09		p = 0.06
**1990–1995**	1		1	
**1995–1999**	0.56 [0.26, 1.23]		0.57 [0.26, 1.25]	
**2000–2004**	0.62 [0.34, 1.15]		0.62 [0.33, 1.15]	
**2005–2007**	0.39 [0.18, 0.86]		0.37 [0.17, 0.80]	

† Adjusted by all other factors in the model.

## Discussion

There are relatively few long-term observational studies of HIV discordant couples in the pre-ART era. Strengths of this study include the availability of data since 1990, before many interventions became widely available to the population, and the observational study design rather than a trial population, which is likely to be generalisable. This study found a high HIV incidence rate in HIV serodiscordant couples (7.11/100 PYAR), and incidence was twice as high in females as in males.

HIV negative partners in steady HIV serodiscordant partnerships are at high risk for HIV acquisition if the HIV positive partner is not on ART. The HIV rate in this study is comparable to those reported from HIV serodiscordant couples elsewhere in sub-Saharan Africa (range 4–10/100 PYAR) [6], [8]–[12]. In contrast, the highest recorded annual HIV incidence in the general study population from which these couples were drawn during the period between 1990 and 2004 was 0.8/100 PYAR [19], [20]. A previous study in this population found that the rate ratios for serodiscordant versus concordant negative couples were 11.6 in HIV negative men and 105.8 in HIV negative women [6]. These results highlight that urgent efforts are needed to identify discordant couples through increased uptake of counselling and testing, by ensuring that services are widely available and accessible for couples [26].

The main factors associated with HIV transmission within a couple were a male index partner, non-Muslim couple, high viral load in the index partner, and a greater age difference between spouses. The median age at HIV seroconversion was substantially higher in men (40 years) than in women (28 years), and this is likely to partly reflect the fact that in this population men tend to be older than their female partners. However, this difference was also observed in the general population from which the cohort came, in which median age at seroconversion was higher in men [19], likely due to the increased risk of HIV acquisition in females than males due to greater biological susceptibility discussed further below. Additionally, young women tend to have sex with older men who are more likely to be at higher risk through multiple partnerships [27]. Among serodiscordant couples, the overall median age at seroconversion was older than in the general population, which may reflect selection bias, as we have excluded couples who are seroconcordant positive. However, these findings highlight that the older age at seroconversion provides an opportunity for prevention in younger discordant couples. Further, following the first known HIV status for the couple, the median duration of follow-up before seroconversion was 2 years, however more frequent testing and counselling of couples is likely to identify couples in whom HIV discordance is recent. This would then provide an opportunity for risk reduction and prevention of transmission.

HSV-2 infection in the HIV negative partner was associated with a doubling of the rate of seroconversion (although the confidence intervals were wide). This magnitude of association is consistent with a meta-analysis of 25 cohort studies in which prevalent HSV-2 increased the risk of HIV acquisition three-fold (adjusted RR 2.8 (95% CI 2.1–3.7) in men and 3.4 (95% CI 2.4–4.8) in women) [28]. There is good biological plausibility for an association between HSV-2 infection and HIV. HSV-2 is known to cause breakages of the genital mucosa and thereby increase the risk of entry of HIV. In addition HSV-2 recruits HIV target cells in the genital mucosa thereby increasing the risk of HIV infection [29]–[31]. However the increased risk of HIV acquisition associated with HSV-2 infection seen in epidemiologic studies may be partly due to unmeasured confounding from high risk behaviour given that both infections are acquired sexually [32]. Despite this evidence, two RCTs of HSV-2 suppressive treatment found no evidence of a reduction in HIV incidence rates [33], [34]. It is possible that the dosage given (twice daily 400 mg of acyclovir) may have been inadequate to achieve sufficient suppression of HSV-2 to prevent HIV acquisition. Also, adherence to acyclovir in these trials may have been sub-optimal.

HSV-2 infection in the positive partner was associated with a slightly lower rate of HIV transmission to the HIV negative partner (adjusted HR 0.61 [95% CI: 0.24, 1.57], p = 0.4). The result was unexpected as HSV-2 is thought to increase the infectiousness of HIV in co-infected persons. Previous observational studies of the association of HSV-2 with HIV incidence in discordant couples have also been inconclusive, with one study [35] reporting no increase in risk of transmission in association with HSV-2 infection and the other [36] reporting more frequent diagnosis of HSV-2 among seroconverting couples compared to couples remaining serodiscordant (46.2% vs 3.6%) after 6 to 12 months of follow up.

Over half the index partners in this study were male (54%). This is in contrast to the Partners in Prevention study from 7 eastern and southern African countries, in which 33% of index partners were male [37]. Men were older than women in this study population and therefore more likely to have prevalent HIV. In addition, it is possible that HIV positive men are more likely to remarry e.g. after separation of death of a spouse, than HIV positive women. However, HIV incidence in the cohort was higher among women. Women might be at higher risk of HIV incidence because of the larger mucosa area in the vagina than the male foreskin, and because the low vaginal pH is hostile to HIV therefore vaginal secretions may carry less virus than semen thereby potentially rendering women to be less infectious than men, and further, semen increases vaginal pH thereby rendering it less hostile to the HIV. In addition, semen stays in the vaginal column for longer than vaginal secretions stay on the penis, and so women may have longer exposure to infection and therefore be at higher risk [1], [38].

The rate of HIV transmission was higher when the man was older by more than 15 years, especially if the HIV negative partner was female. Similar results were seen in a longitudinal study in Zimbabwe that reported increased vulnerability to HIV in young women who have sexual relationships with older, and usually high risk men [27]. One explanation for this is that younger women (and those in relationships with older men) are more likely to engage in extramarital sex, and hence the HIV infection is externally acquired. Younger women may also be vulnerable because of larger area of cervical ectopy as compared to older women, and lack of power to negotiate safer sex with their partners [39].

Muslims were at a significantly lower risk of HIV acquisition in our study (aHR = 0.27, 95%CI 0.11–0.68) presumably because of the almost universal practice of male circumcision among Muslims in this population. There is little evidence that male circumcision directly reduces risk of male to female HIV transmission [40], [41] but because marriages tended to be between partners of the same religion, Muslim women may have had lower risk owing to lower incidence rates in the male partners for extramarital infection.

Rates of HIV seroconversion reduced over time. During the early period (1990–1994), there was low awareness of one’s own, or partners’, HIV status. Counselling advice e.g. for the use of condoms in the context of serodiscordant partnerships was also not widely available [42]. As a consequence, little was done to prevent HIV transmission in marriage or longstanding sexual partnerships. Counselling and testing for HIV, condoms, treatment of opportunistic infections and antiretroviral treatment have become increasingly available in recent years and are likely to explain the reduction in seroconversion rates over time. No seroconversions occurred among couples in which the HIV positive partner was on HAART. Reduced risk of HIV transmission in the present of HAART has been reported in other observational studies [16], [43] and recently confirmed in a randomised clinical trial [15].

This study had a number of limitations. Firstly it was not ascertained whether HIV seroconversions occurred as a result of transmission from within the partnership or from an external partner. Genetic sequencing of couples’ virus has found up to 30% non-matching virus indicating infection acquired outside of the partnership [13], [44]. Therefore the rates of seroconversion reported in this study may be higher than within-couple transmission rates. Secondly, average coverage for the annual survey was about 60%, and we further excluded couples with incomplete HIV status and couples seen once only. The couples included in the incidence analysis were more likely to have a female negative partner than those seen once only, and this may have resulted in a higher estimate of overall HIV seroconversion than would be expected in this population. However, our estimated incidence is comparable to estimates from serodiscordant couple studies in neighbouring populations [8]–[12]. Finally, we did not have data on other STIs, knowledge of own or partners HIV status, and had relatively few data on viral load and CD4 count. As a consequence, we may have failed to measure the potentially confounding effect from these factors. For example knowledge of one’s own HIV status or that of the partner may influence behaviour and cause one to adopt preventive measures including abstinence, condom use, seeking counselling, or treatment thereby reducing risk of HIV transmission despite their HSV-2 status. We did not have data on condom use. However condom use in the context of stable partnership is rare.

### Conclusions

HIV negative partners in serodiscordant couples have a high incidence of HIV infection if the index partner is not on anti-retroviral therapy. Before these become available, there should be continued emphasis on couples counselling and testing (for example within the programmes of increased voluntary medical male circumcision scale-up), and HIV serodiscordant couples should be strongly advised to use the existing interventions to minimise risk of HIV transmission.
